# GNSS smartphones positioning: advances, challenges, opportunities, and future perspectives

**DOI:** 10.1186/s43020-021-00054-y

**Published:** 2021-11-16

**Authors:** Farzaneh Zangenehnejad, Yang Gao

**Affiliations:** Department of Geomatics Engineering, Schulich School of Engineering, University of Calgary, 2500 University Drive N.W, Calgary, AB T2N 1N4 Canada

**Keywords:** Smartphone positioning, GNSS, Carrier-to-noise density ratio (C/N_0_), Precise point positioning (PPP), Real-time kinematic positioning (RTK)

## Abstract

Starting from 2016, the raw Global Navigation Satellite System (GNSS) measurements can be extracted from the Android Nougat (or later) operating systems. Since then, GNSS smartphone positioning has been given much attention. A high number of related publications indicates the importance of the research in this field, as it has been doing in recent years. Due to the cost-effectiveness of the GNSS smartphones, they can be employed in a wide variety of applications such as cadastral surveys, mapping surveying applications, vehicle and pedestrian navigation and etc. However, there are still some challenges regarding the noisy smartphone GNSS observations, the environment effect and smartphone holding modes and the algorithm development part which restrict the users to achieve high-precision smartphone positioning. In this review paper, we overview the research works carried out in this field with a focus on the following aspects: first, to provide a review of fundamental work on raw smartphone observations and quality assessment of GNSS observations from major smart devices including Google Pixel 4, Google Pixel 5, Xiaomi Mi 8 and Samsung Ultra S20 in terms of their signal strengths and carrier-phase continuities, second, to describe the current state of smartphone positioning research field until most recently in 2021 and, last, to summarize major challenges and opportunities in this filed. Finally, the paper is concluded with some remarks as well as future research perspectives.

## Introduction

In May 2016 and during the “Google I/O” conference, Google announced that the raw GNSS measurements, i.e., the pseudorange, carrier-phase, Doppler shift and carrier-to-noise density ratio (C/N_0_) observations, would be accessible through the Android Nougat (version 7) operating systems. In August 22, 2016, the Android 7 (Nougat) was officially released by Google which can be regarded as a breakthrough for the GNSS community. Since then, many researches have been conducted to develop new algorithms to improve the performance of GNSS positioning using these mass-market devices. Early smartphones only provided single-frequency and mostly GPS-only observations. In 2017, the Samsung S8 and Huawei P10 smartphones were released as the first multi-GNSS devices which are able to track carrier-phase measurements. However, in May 2018, the Xiaomi Mi 8 equipped with the new Broadcom BCM47755 GNSS chipset was released as the world’s first dual-frequency GNSS smartphone, i.e., added with L5 for GPS and QZSS and E5a for Galileo (European GNSS Agency, GSA, 2018a). It can be also regarded as a great millstone in smartphone positioning as it provides the users with an opportunity to make ionospheric-free linear combination between observations of two frequencies to eliminate the ionosphere effect.

Following the release of the BCM47755 by Broadcom, other key chipset manufacturers have also developed the dual-frequency chipsets such as Qualcomm with the Snapdragon X24 LTE modem and HiSilicon with the Kirin 980 system-on-a-chip (GPS World, 2018). In late 2019, Qualcomm Technologies, Inc. announced a collaboration with Indian Space Research Organization (ISRO) to help provide chipset platforms which support India’s Regional Navigation Satellite System (IRNSS) for the first time. The initiative will help improve the location positioning accuracy and robustness of location-based services in the region (Cozzens, 2020a). The BCM47765 has also been introduced by Broadcom in May 2020 as the second-generation dual-frequency GNSS solution capable of tracking the new BeiDou Navigation Satellite System (BDS-3) constellation’s B2a signals. The BCM47765 simultaneously supports Global Positioning System (GPS), GLObal NAvigation Satellite System (GLONASS), Navigation with Indian Constellation (NavIC), BeiDou Navigation Satellite System (BDS), Galileo Navigation Satellite System (Galileo), Satellite-Based Augmentation Systems (SBAS), and Japanese Quasi‑Zenith Satellite System (QZSS) in both the L1/ B1/E1 and L5/E5a/B2a frequency bands. This has led to improved availability (30 new L5 signals which is about 60% more) and accuracy (Cozzens, 2020b).

Currently there are several hundreds of smartphone models on the market capable of providing the raw GNSS observations. A list of available devices capable of providing GNSS raw measurements including constellation availability and frequencies they track can be found at the following website [Accessed 17 August 2021] https://docs.google.com/spreadsheets/d/1z6Yt9c4cyev1PB6VWEkbZtJGfoxAQ5UJnHyP24sFwlk/edit#gid=0

which was created by Dr. Sean Barbeau who is the principal mobile software architect for R&D at University of South Florida and the developer of the GPSTest app. GPSTest app is an open-source GNSS testing app providing positioning data to your device (e.g., GPS, GLONASS, Galileo, BDS, QZSS, Indian Regional Navigation Satellite System (IRNSS) and SBAS).^1^

**Fig. 1:**
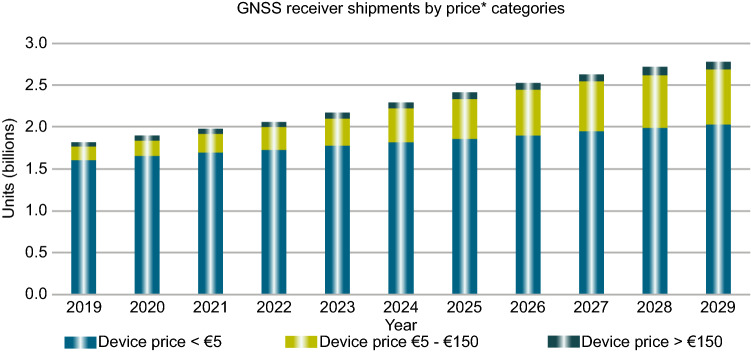
Global annual GNSS receiver shipments from 2019 to 2020 (European GSA, 2019a)

Shown in Fig. 1 are the global annual GNSS receiver shipments from 2019 to 2020 (European GSA, 2019a). As can be seen, a majority of shipments is related to the mass-market receivers that are costing less than €5. They are mostly employed in smartphones and wearables. This figure indicates the importance of the research on smartphone positioning, as it has been doing in recent years. Paziewski (2020) gave a review of the recent advances and perspectives for positioning and applications with smartphone GNSS observations. The aim of this paper is also to provide an overview of the research works carried out in this field with a focus on the following aspects:

- to provide a review of fundamental materials on how to obtain GNSS measurements through the new Location API namely android.location operated on Android version 7 or higher. We then summarize available apps which can be used to log the raw GNSS observations as well as the sensor data (accelerometer, gyroscope and magnetometer). They are of value to researcher and engineers who are developing precise positioning algorithms and products with smartphones GNSS observations.
- to provide a review of fundamental work on quality assessment of raw GNSS smartphone measurements from different smartphones in terms of their signal strengths and the carrier-phase continuity. The data that have been analyzed here is a part of a competition hosted by Google called "Google Smartphone Decimeter Challenge" (Fu et al., 2020). It gives the reader a great insight into the quality of smartphone observations and the accuracy-level that one can expect.
- to describe the current state of smartphone positioning research field including the most recent work in 2021.
- to provide an overview of most recent efforts and progresses in the field of smartphone GNSS positioning and applications and a summary of major challenges and opportunities in this filed.

This review paper is organized as follows. In the next section, a review of researches on GNSS smartphone positioning and applications carried out before the release of Android 7 is provided. With the release of Android 7, one can access the raw GNSS measurements; how to access to is therefore briefly explained. A list of GNSS android loggers is also provided. In continue, the performance of different smartphones is investigated use is made of the datasets provided by Google for the Google Smartphone Decimeter Challenge (Fu et al., 2020). After that, a review of literatures is provided by summarizing major recent contributions devoted to single and precise point positioning, relative positioning as well as GNSS/INS (Inertial Navigation System) integration employing the smartphones measurements. Finally, we summarize some major challenges and opportunities in the field of smartphone positioning by highlighting future research perspectives.

## GNSS smartphone positioning and applications

A literature review on GNSS smartphone positioning and applications is provided in this section, which is divided into two main parts, each of which covers a specific time period (before the release of the Android 7 and after the release of the Android 7).

## Researches before the releases of the Android 7 on May 2016

Prior to 2016, only the position-velocity–time (PVT) computed by the GNSS chipsets was available to the users and the raw GNSS observations (e.g., pseudorange and carrier-phase measurements) were not available. The positioning accuracy of a GNSS module on a smartphone was typically between 3 and 5 m under good multipath conditions and over 10 m under harsh multipath environments (Bi et al., 2016). Such an accuracy level was not good enough for some applications. Therefore, several efforts were made to improve the positioning accuracy using the GNSS smartphones. Since the raw GNSS measurements were not accessible, improving the smartphone GNSS positioning accuracy was not possible without using the external hardware or the user-developed software (Yoon et al. 2016).

Hwang et al. (2012) developed a smartphone application with wireless communication, NTRIP (networked transport of RTCM via internet protocol) client, and real-time kinematic (RTK) processing features that simplify the network RTK (NTRK) and reduce the required cost. The smartphone application could provide differential GNSS (DGNSS) or RTK corrections in the coordinate domain to a GNSS receiver connected to a smartphone via Bluetooth. As a result, the positioning accuracy could be improved. Byungwoon et al. (2013), Park et al. (2013) and Chen et al. (2014) also proposed a method in which the pseudorange corrections could be converted to the coordinate corrections in the position domain (i.e., differential GNSS in coordinate domain or DGNSS-C). Therefore, one could improve the initial coordinates obtained from the smartphone GNSS chipset by applying the coordinate corrections without any need to have access to the raw pseudorange data.

Pesyna et al. (2014) investigated the performance of a GNSS smartphone antenna connected to an external radio frequency (RF) front-end and GNSS receiver. They used a smartphone antenna to direct the GNSS signals into a software-defined receiver generating the carrier-phase and pseudorange observations. The double-difference (DD) pseudorange and phase observations were then post-processed. They, for the first time, showed that cm-level accuracy can be achieved using a smartphone-quality GNSS antenna in relative positioning mode. They also demonstrated that the smartphone GNSS observations are highly affected by the multipath error which is due to the fact that the smartphone antenna is linearly polarized (instated of circularly polarized in geodetic antennas) making it more vulnerable to the multipath effects. This, in turn, leads to more difficult carrier-phase ambiguity resolution. Even though the results were impressive, the research was not practical since it was only limited to the external RF front-end and processing software instead of real smartphone observations collected by a GNSS chip embedded in a smartphone.

Kirkko-Jaakkola et al. (2015) were investigated the quality of raw GNSS measurements of a Nokia Lumia 1520 smartphone using custom firmware allowing access to the raw GNSS measurements from the phone’s internal GNSS receiver. They also compared the positioning performance of the Nokia Lumia 1520 smartphone with a U-Blox receiver. The results showed that the smartphone measurements were nosier than the U-Blox ones. They were also contaminated by multiple outliers in comparison with the U-Blox receiver observations. The authors also assessed the performance of both Nokia Lumia 1520 smartphone and U-Blox receiver with respect to the RTK solution. The results indicated that a meter-level positioning accuracy was achieved employing the Nokia Lumia 1520 GPS-only observations.

Along with the Google announcement in May 2016 regarding the availability of the raw GNSS measurements from the smartphones or tablets running the Android version 7 and higher, it became possible to develop advanced algorithms to improve the positioning accuracy of the ultra-low-cost GNSS smartphones. Therefore, the next section provides the most researches done after such a great advance.

## Researches after the releases of the Android 7

After the release of the Android Nougat (version 7) operating systems, raw GNSS measurements from smartphones and tablets became accessible. This then provides the researchers and developers with an opportunity to develop new algorithms for improving the performance of smartphone positioning. This section consists of three subsections, first availability of the raw GNSS measurements will be explained. Next, we assess the quality of GNSS observations from four smart devices in terms of the C/N_0_ and carrier-phase continuity. Finally, we will provide most contributions devoted to the single/precise point positioning algorithm development, the relative positioning method and the GNSS/INS integration using the smartphones measurements.

### Access to raw GNSS measurements

**Fig. 2:**
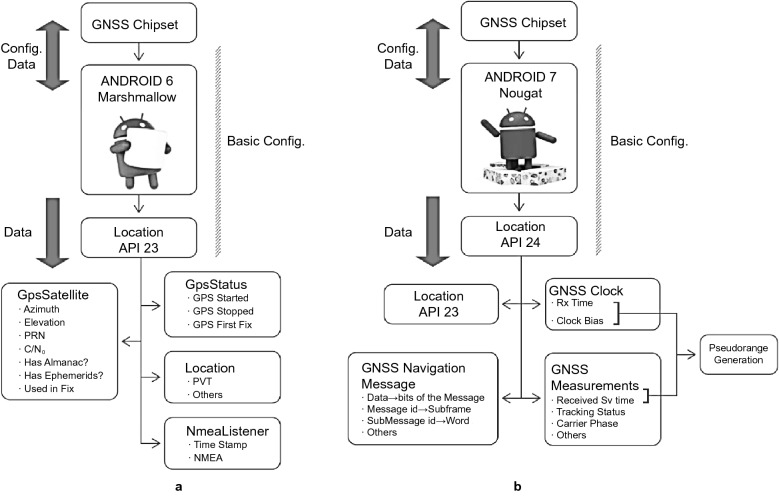
Location API in **a** Android version 6 (Marshmallow) and **b** Android version 7 (Nougat) (European GSA, 2018b)

The Android system provides a series of functions called application programming interface (API) allowing users to access the systems functionalities. Each version of the Android system has different types of APIs. Before releasing the Nougat version of the Android system in 2016, location information was available through the android.gsm.location API providing only some basic information such as GPS satellite information (e.g., C/N_0_, azimuth, and elevation) as well as the basic NMEA (National Marine Electronics Association) sentences which include the PVT solution (Fig. 2a). Starting from the Nougat version (Version 7), Android introduces the new Location API namely android.location. However, the new API implemented on Android 7 does not still provide the GNSS measurements directly (e.g., pseudorange, carrier-phase and Doppler observations) but the GNSS measurements needed to extract from the raw data logged (Fig. 2b). A list of variables from GNSSClock and GNSSMeasurement classes within the Android API package “location” is summarized in Tables 1 and 2, respectively.

**Table 1:** List of variables from GNSSClock class within the Android API package “location” (European GSA, 2018b)

Field	Description
*TimeNanos*	GNSS receiver’s internal hardware clock value in nanoseconds
*TimeUncertaintyNanos*	1-Sigma uncertainty associated with the clock's time in nanoseconds
*FullBiasNanos*	Difference between TimeNanos inside the GPS receiver and the true GPS time since 6 January 1980
*BiasNanos*	Clock’s sub-nanosecond bias
*BiasUncertaintyNanos*	1-Sigma uncertainty associated with the local estimate of GPS time (clock bias) in nanoseconds
*DriftNanosPerSecond*	Clock’s drift
*DriftUncertaintyNanosPerSecond*	1-Sigma uncertainty associated with the clock's drift in nanoseconds (per second)
*HardwareClockDiscontinuityCount*	Count of hardware clock discontinuities
*LeapSecond*	Leap second associated with the clock’s time
*ChipsetElapsedRealtimeNanos*	Time since system boot (Added in API 29)

**Table 2:** List of variables from GNSSMeasurement class within the Android API package “location” (European GSA, 2018b)

Field	Description
*ConstellationType*	Constellation type (GPS: 1, SBAS: 2, GLONASS: 3, QZSS: 4, BDS: 5, Galileo: 6, Unknown: 9)
*Svid*	Satellite ID (GPS: 1, SBAS: 2, GLONASS: 3, QZSS: 4, BDS: 5, Galileo: 6, Unknown: 9)
*TimeOffsetNanos*	Time offset at which the measurement was taken in nanoseconds
*State*	Current state of the GNSS engine
*ReceivedSvTimeNanos*	Received GNSS satellite time at the measurement time
*ReceivedSvTimeUncertaintyNanos*	1-Sigma uncertainty of the Received GPS Time-of-Week in nanoseconds
*Cn0DbHz*	Carrier-to-noise density in dB-HZ in the range [0,63]
*PseudorangeRatemetersperSecond*	Gets the Pseudorange rate at the timestamp in m/s
*PseudorangeRateUncertaintyMetersPerSecond*	1-Sigma uncertainty of the pseudorange_rate_mps
*AccumulatedDeltaRangeState*	Validity of the carrier measurements as follows ADR_STATE_CYCLE_SLIP:4 ADR_STATE_RESET: 2 ADR_STATE_VALID: 1 ADR_STATE_UNKNOWN: 0 Note: Only valid measurements should be used for calculation
*AccumulatedDeltaRangeMeters*	Accumulated delta range since the last channel reset
*AccumulatedDeltaRangeUncertaintyMeters*	1-Sigma uncertainty of the accumulated delta range in meters
*CarrierFrequencyHz*	Carrier frequency of each tracked signal in Hz
*CarrierCycles*	Number of full carrier cycles between the satellite and the receiver (Deprecated in API level 28)
*CarrierPhase*	RF phase detected by the receiver (Deprecated in API level 28)
*CarrierPhaseUncertainty*	1-Sigma uncertainty of carrier-phase (Deprecated in API level 28)
*MultipathIndicator*	A value indicating the 'multipath' state of the event
*SnrInDb*	Signal-to-noise ratio at correlator output in dB
*AgcDb*	Automatic gain control (AGC) level
*BasebandCn0DbHz*	Baseband carrier-to-noise density in dB-Hz (Added in API level 30)
*FullInterSignalBiasNanos*	GNSS measurement's inter-signal bias in nanoseconds with sub-nanosecond accuracy (Added in API level 30)
*FullInterSignalBiasUncertaintyNanos*	1-Sigma uncertainty of GNSS measurement's inter-signal bias in nanoseconds (Added in API level 30)
*SatelliteInterSignalBiasNanos*	GNSS measurement's satellite inter-signal bias in nanoseconds with sub-nanosecond accuracy (Added in API level 30)
*SatelliteInterSignalBiasUncertaintyNanos*	1-Sigma uncertainty of GNSS measurement's satellite inter-signal bias in nanoseconds (Added in API level 30)
*CodeType*	GNSS measurement's code type (Added in API level 29)

In the next subsection, we briefly provide some explanations about how to obtain GNSS time, pseudoranges, carrier-phase and Doppler measurements. The reader may refer to the white paper published by the European GNSS Agency’s (GSA) to find more details (European GSA, 2018b).

#### GPS time generation

The GPS reference time in nanoseconds can be obtained using the internal hardware clock and the bias to the true GPS time as follows:

1 $$GPS time\, =\, TimeNanos - \left( {FullBiasNanos + BiasNanos} \right)$$

when the receiver has estimated the time using GPS time. If the receiver has estimated the time using a non-GPS constellation, Eq. (1) becomes as follows

2 $$GPS time\, =\, TimeNanos - \left( {FullBiasNanos + BiasNanos} \right) - InterSystemBias$$

where $InterSystemBias$ is the offset between GPS time and the GNSS time used in the time estimation.

#### Pseudorange observation generation

The pseudorange observations can be obtained using the time difference between the received time (measurement time) $t_{Rx}$ and the transmitted time $t_{Tx}$ as follows:

3 $$P = \left( {t_{Rx} - t_{Tx} } \right) \times 10^{ - 9} \times c$$

where $P$ is the pseudorange observation in meter, $t_{Tx} = ReceivedSvTimeNanos \left[ {ns} \right]{ }$ is the received GNSS satellite time at the measurement time in nanosecond (i.e., the GNSS reference time when the signal was transmitted) and $c = 299792458.0 \left[ {m/s} \right]$ is the speed of light. One can then obtain the measurement time $t_{Rx}$ in GNSS time system in nanosecond as follows:

4 $$t_{Rx GNSS} = TimeNanos + TimeOffsetNanos - \left( {FullBiasNanos\left( 1 \right) + BiasNanos\left( 1 \right)} \right)$$

It should be noted that only the first value of $FullBiasNanos$ and $BiasNanos$ is used to compute all the received times (i.e., $FullBiasNanos\left( 1 \right)$ and $BiasNanos\left( 1 \right)$) as long as there is no discontinuity in the internal received time. In Eq. (4), both $t_{Rx GNSS}$ and $t_{Tx}$ must be in the same time system for all GNSS systems. $t_{Rx GNSS}$ is provided in the GNSS reference system while $t_{Tx}$ is provided for each GNSS system (for example GPS time or GLONASS time and etc.). Therefore, one must convert to other one (i.e., same GNSS time system). Usually, GPS time (GPST) is implemented as the default reference time. Another important point is that $t_{Rx GNSS}$ and $t_{Tx}$ must be in the same range which depends on the tracking status which is represented by “*State*” in Table 2. Table 3 provides a summary of how to compute the measurement time $t_{Rx}$ for each GNSS time system depending on the tracking status. All values in the equations are in nanosecond. In this table, *mod* is remainder after division (modulo operation), $NumberNanoSecondsWeek = 604800{\text{e}}9$ is the number of nanoseconds within one week, $NumberNanoSecondsDay$ is the number of nanoseconds within one day and $NumberNanoSeconds100Milli$ is the number of nanoseconds within 100 ms. After computing $t_{Rx}$, one can compute the pseudorange from Eq. (3).

**Table 3:** Summary of $t_{Rx}$ [ns] computation

*GPS and Galileo with Time of Week (TOW) decoded status*	$t_{Rx} = mod\left( {t_{Rx GNSS} ,NumberNanoSecondsWeek} \right)$
*BDS with TOW decoded status*	$t_{Rx} = mod\left( {t_{Rx GNSS} ,NumberNanoSecondsWeek} \right) + 14 \times 10^{9}$
*Galileo with E1C 2nd status*	$t_{Rx} = mod\left( {t_{Rx GNSS} ,NumberNanoSeconds100Milli} \right)$
*GLONASS with Time of Day (TOD) status*	$t_{Rx} = mod\left( {t_{Rx GNSS} ,NumberNanoSecondsDay} \right) + \left( {3 \times 3600 \times 10^{9} } \right) - Leapsecond \times 10^{9}$

#### Carrie-phase observation generation

As explained in Table 2, it is better to use only valid measurements for the carrier-phase observation calculation. The carrier-phase observation in cycle can be obtained as $\varphi = AccumulatedDeltaRangeMeters/\lambda$ where $\lambda$ is the signal’s wavelength. It should also note that they are ambiguous, meaning the receiver can only count the number of cycles occurring between epochs.

#### Doppler observation generation

The Doppler shift causing from the satellite movement can be obtained as follows

5 $$dpplershift = - PseudorangeRatemetersperSecond/\lambda$$

### Exiting apps

In 2016, Google released an open source application namely “GnssLogger” app that logs the measurements described in the GnssClock and GnssMeasurement classes from the android.location API documentation. It is available at (Accessed 17 August 2021).

https://developer.android.com/training/location/

At first, this app only provided the android.location API raw measurements in CSV format including all types of location and sensor data such as GNSS and other sensor data. However, in the updated version (v3.0.0.1), the GNSS observations can be directly saved in RINEX format as well. It is also capable of logging sensor data such as accelerometer, gyroscope and magnetometer data.

Other GNSS logger Android applications have also been developed later. A list of GNSS android loggers is provided in Table 4. They are as follows:

- GNSSLogger app: It was released by Google in 2016. The output format is either CSV, RINEX or NMEA (van Diggelen & Khider, 2018). GNSS logger is also capable of logging GNSS and sensor data (accelerometer, gyroscope and magnetometer) from the smartphones.
- Geo++ RINEX Logger app: It was released by the Geo++ GmbH Company in 2017 capable of providing GNSS observables in RINEX format (Geo++ GmbH, 2018).
- rinexON app: It was released by FLAMINGO team in 2018 capable of providing GNSS observables in RINEX format (Nottingham Scientific Ltd, 2018).
- GalileoPVT app (Crosta & Watterton, 2018): It was released by the European Space Agency (ESA). The raw measurements can be logged in CSV or NMEA format.
- G-RitZ logger (Kubo, 2018): It has been developed at Ritsumeikan University. The app aim to output in RINEX format.
- GNSS/IMU Android Logger: It has been recently developed at Universität der Bundeswehr München. It is capable of logging GNSS data in raw measurement format/RINEX 3.03 and sensor data (accelerometer, gyroscope and magnetometer) from the smartphone simultaneously (Sharma et al. 2021).

**Table 4:** Available GNSS logger Android applications

App	Developer	Output format
*GNSSLogger app*	Google	CSV, NMEA and RINEX
*Geo*++ *RINEX Logger app*	Geo++ GmbH Company	RINEX
*rinexON app*	FLAMINGO	NMEA, RINEX
*GalileoPVT app*	European Space Agency (ESA)	CSV and NMEA
*G-RitZ logger*	Ritsumeikan University	NMEA, RINEX
*GNSS/IMU Android Logger*	Universität der Bundeswehr München	CSV, RINEX and IMU data

### Smartphone GNSS observations quality assessment using real observations

The quality of smartphone GNSS observations plays an important role in high-precision smartphone positioning. Before starting the next section and providing the literature review devoted to the recent advances in the field of smartphone positioning, we assess the characteristics of GNSS observations from several GNSS smartphones in terms of their C/N_0_ records and carrier-phase continuity. The C/N_0_ referred to the ratio of the carrier power and the noise power per unit bandwidth. It can be regarded as a powerful indicator of the GNSS signal strength in the sense that a larger C/N_0_ indicates a stronger signal while a lower C/N_0_ shows a weaker signal.

Observations of four GNSS smartphones namely Google Pixel 4, Google Pixel 5, Xiaomi Mi 8 and Samsung Ultra S20 are used. The first two devices use the Qualcomm chipset while the last two ones use the Broadcom chipset. All four devices are dual-frequency smartphones supporting L5/E5a frequencies for GPS and Galileo, respectively. However, we only focus on GPS, GLONASS and Galileo on the L1 frequency (GPS, GLONASS and Galileo were common constellations between all four devices). The data being used here was collected by the Google team as a part of a competition hosted by Google namely Google Smartphone Decimeter Challenge. They have logged on 2021-03-25 for about 30 min with a sampling interval of 1 s in a kinematic mode in Palo Alto, CA, USA, using the GNSSLogger app with the duty-cycle off. They can be found at g.co/gnsstools.

**Fig. 3:**
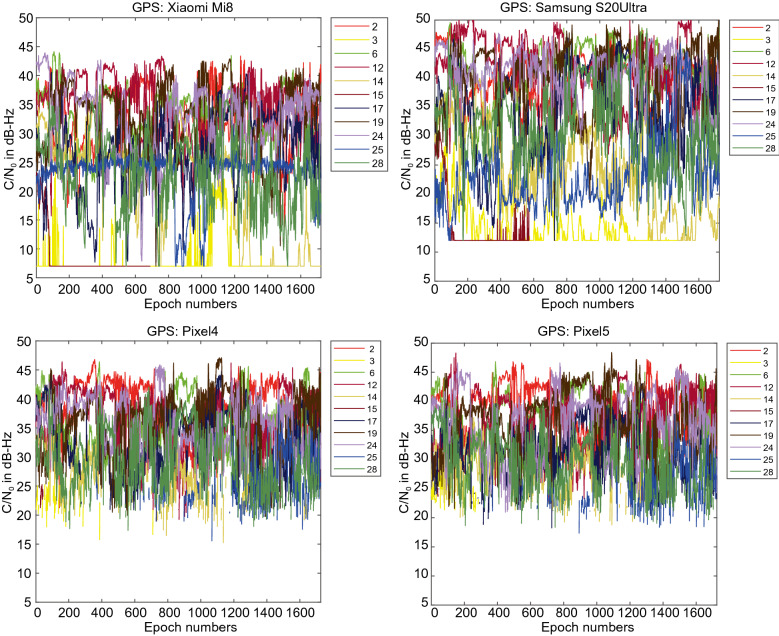
C/N_0_ measurements for GPS L1 signal for all four devices

**Fig. 4:**
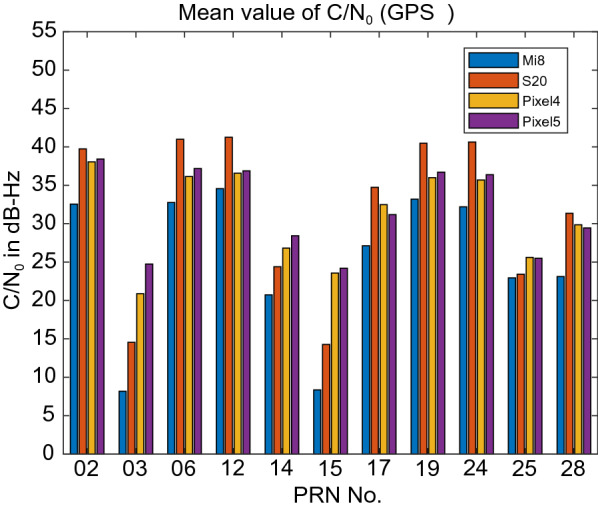
Mean value of C/N_0_ for all GPS PRNs

**Fig. 5:**
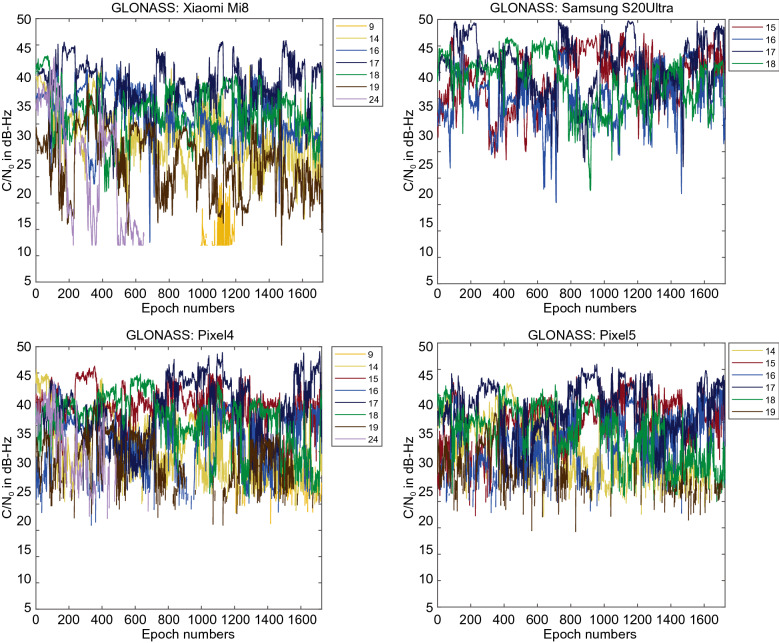
C/N_0_ measurements for GLONASS L1 signal for all four devices

**Fig. 6:**
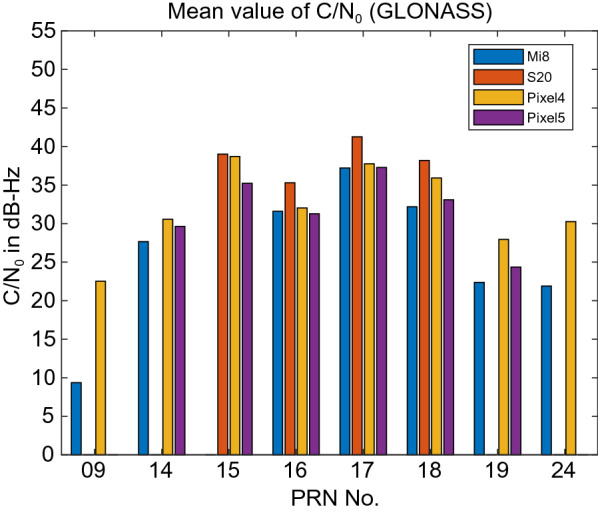
Mean value of C/N_0_ for all GLONASS PRNs

**Fig. 7:**
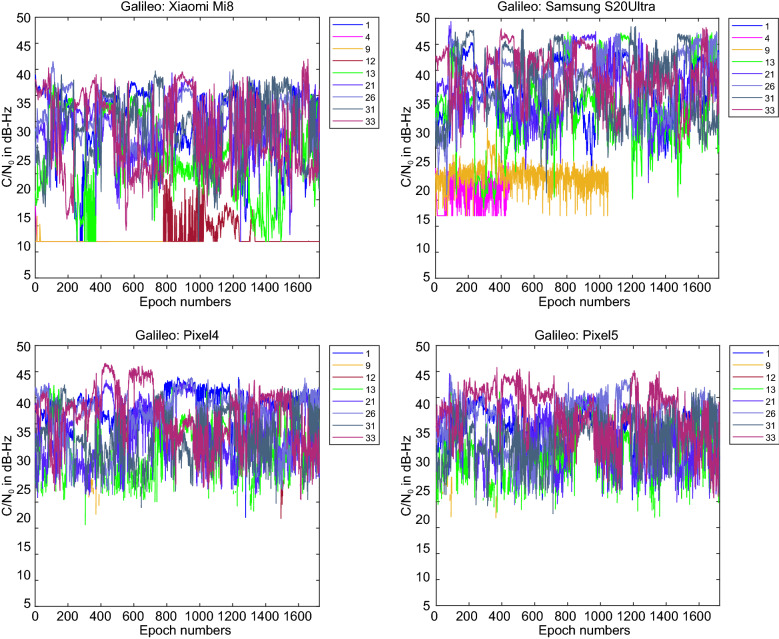
C/N_0_ measurements for Galileo E1 signal for all four devices

**Fig. 8:**
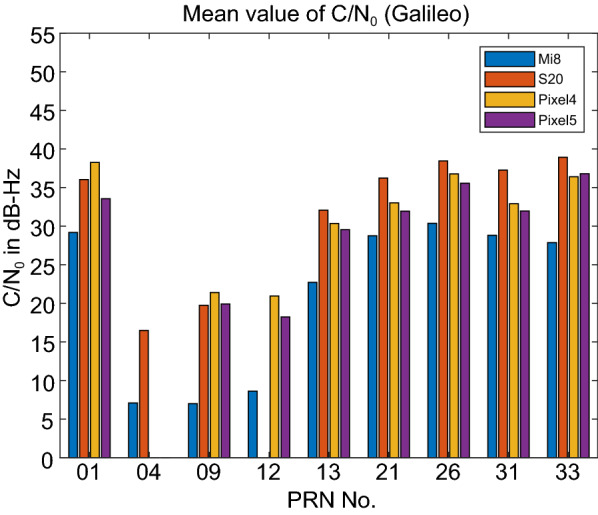
Mean value of C/N_0_ for all Galileo PRNs

Figure 3 provides the C/N_0_ measurements for GPS L1 signal for all four devices. The GPS C/N_0_ ranges from 7–45, 12–50, 15–45 and 17–45 dB-Hz for Xiaomi Mi 8, Samsung Ultra S20, Pixel 4 and Pixel 5, respectively. The results reveal that the Pixel 4 and Pixel 5 have better performance in terms of C/N_0_ consistency (uniformity). Also, they block the signals with low C/N_0_. To have a better comparison, the mean values of C/N_0_ for all PRNs have been provided in Fig. 4. It indicates the lowest record of C/N_0_ belongs to the Xiaomi Mi 8. Figures 5 and 6 also give the C/N_0_ measurements for GLONASS and the mean value of C/N_0_ for all PRNs, respectively. Finally, Figs. 7 and 8 provides the C/N_0_ measurements for Galileo and the mean values of C/N_0_ for all PRNs, respectively. The lowest record of the Xiaomi Mi 8’s C/N_0_ can also be observed for GLONASS and Galileo. In all figures, one can see that the GNSS measurements of the smartphones have rapid changes/variations over such short time duration (about 30 min). Such a phenomenon has also been reported in Li and Geng (2019).

**Fig. 9:**
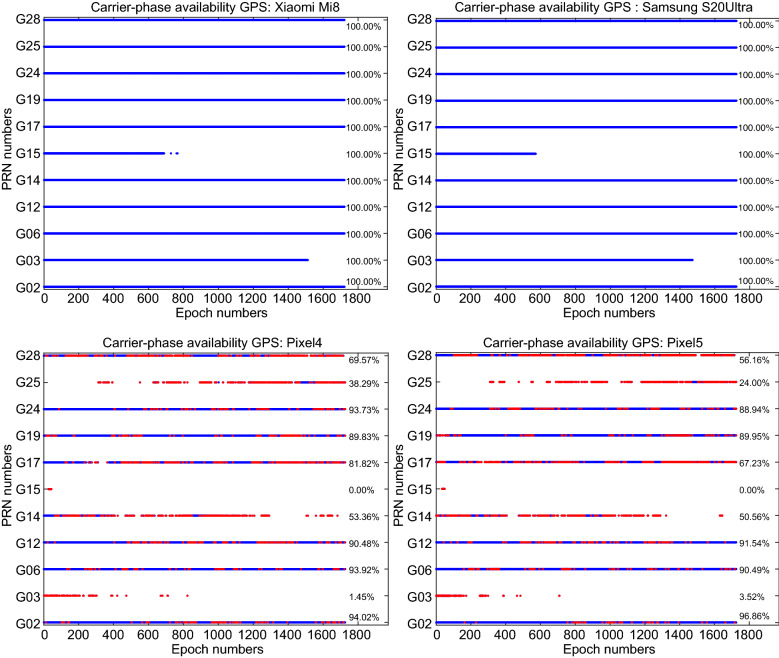
GPS carrier-phase continuity

**Fig. 10:**
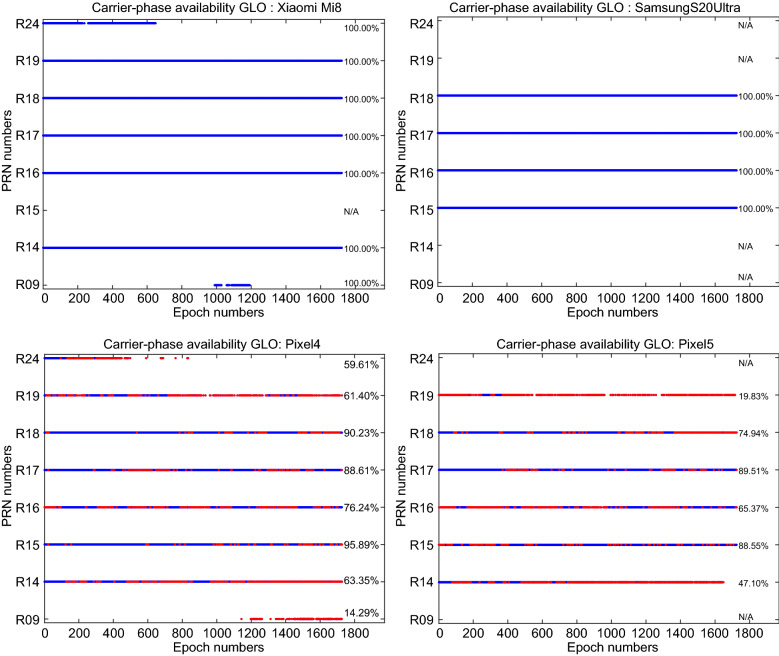
GLONASS carrier-phase continuity

**Fig. 11:**
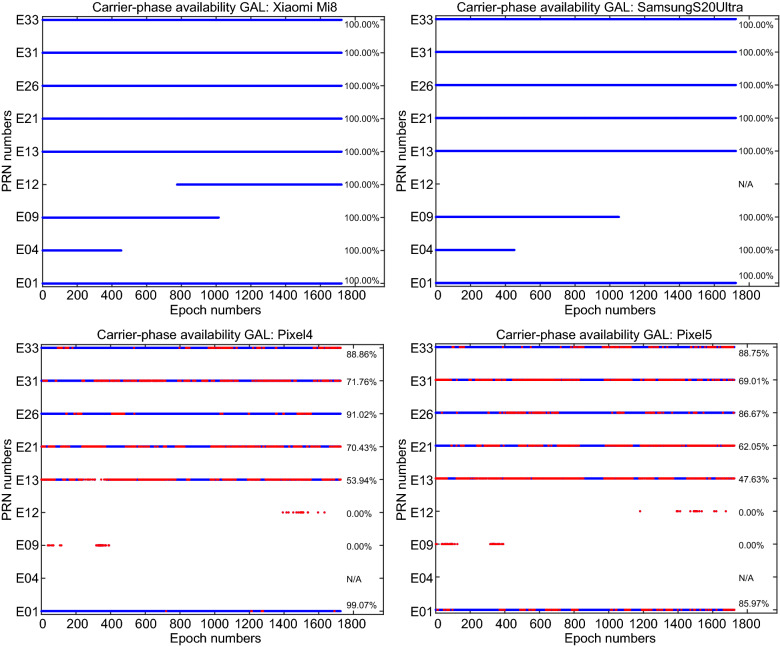
Galileo carrier-phase continuity

In addition to the signal strength indicated by the C/N_0_ values, the continuity (availability) of the GNSS observations is also of importance. Figure 9 provides the GPS carrier-phase continuity for the four devices. In this figure, the red dots denote the epochs in which the carrier-phase observations were missing while the code observations were still observed. This figure indicates that the carrier-phase continuity is preserved for the Xiaomi Mi 8 and Samsung Ultra S20 which can be regarded as a great opportunity for the carrier-phase ambiguity resolution. In Figs. 10 and 11, the same plots are provided for GLONASS and Galileo, respectively. These figures also provide the carrier-phase continuity percentage for each specific satellite.

### Performance assessment of GNSS smartphone positioning

#### Absolute positioning

The content of this subsection summarizes selected recent contributions devoted to single and precise positioning algorithm development applied to smartphone positioning.

Banville and Van Diggelen (2016) conducted the first investigation on the quality of the real raw GNSS observations from the smartphones with the purpose of high-precision positioning. They analyzed the data collected by a Samsung Galaxy S7 smartphone equipped with the Broadcom 4774 GNSS chip which is able to log raw multi-GNSS (GPS, GLONASS, BDS, Galileo, and QZSS) observations on the L1 frequency. However, Banville and Van Diggelen (2016) only employed the GPS observations. Since the true position of the smartphone is unknown, they estimated the positioning errors for all components with respect to the mean values of each component. The results indicated that the pseudorange observations are noisy and only capable of providing meter-level accuracy. They also mentioned that the carrier-phase observations from the smartphones can potentially provide an opportunity to achieve decimeter-level or better positioning accuracy. However, to obtain high-accuracy positioning, some important issues such as the quality of the smartphone antenna and the GNSS duty-cycling, a battery saving mode for the GNSS chip causing discontinuities in carrier-phase observations, must be taken into account.

Navarro-Gallardo et al. (2017) investigated the quality of the raw smartphones measurements and compared the different GNSS constellations with special emphasis on Galileo. Lachapelle et al. (2018) compared the performance of a hand-held GNSS Garmin GPSMap 66 unit with a Huawei P10 smartphone under different conditions including on a roof top of a building, an urban canyon, indoors and in a car. The results indicated a relatively better performance of the GPSMap 66 with respect to the Huawei P10 which is due to the lower gain advantage of the GPSMap 66 over the P10. It was also shown that the use of an external geodetic antenna can significantly improve data quality and positioning accuracy. Gogoi et al. (2018) assessed the smartphone positioning accuracy in a controlled-environment anechoic chamber to mitigate the multipath error allowing them to investigate the duty-cycle phenomena. As they expected, the quality of the observations collected in the anechoic chamber is significantly better than those collected in the real environment. The results showed the noise of pseudorange and carrier-phase observations increased after the duty-cycle occurs. It should note that the option of turning off the duty-cycle is now added to the latest Android release systems. Zhang et al. (2018) first investigated the quality of the raw smartphone observations and draw the same conclusions as the other researchers about the relatively low quality of the smartphone GNSS observations. They also showed that C/N_0_ value of GNSS raw observations of the smartphones is 10 dB-Hz lower than the C/N_0_ values obtained from a geodetic-quality antenna and receiver. They then combined the pseudorange, carrier-phase and Doppler observations by a time-difference (TD) filter positioning algorithm. In this method, they used the Doppler observations to obtain the velocities and then combined them with the single point positioning (SPP) solutions to achieve the sub-meter-level positioning accuracy. Later, Liu et al. (2019) conducted a comprehensive research on the quality of raw GNSS observations of smartphones in terms of the C/N_0_, noise, tracking capability of the carrier-phase, and velocity estimation. Based on the authors’ experience, there was a stronger correlation between the pseudorange accuracy and C/N_0_ rather than the satellite elevation angle. Therefore, an elevation-dependent weighting is not proper for low-cost receivers while a C/N_0_ weighting would be a better choice for these devices. Banville et al. (2019) also suggested a C/N_0_ weighting model instead of an elevation-dependent weighting model. Employing the C/N_0_ weighting model instead of an elevation-dependent weighting model has been also reported by Banville et al. (2019), Liu et al. (2019), Paziewski et al. (2019) and Robustelli et al. (2021).

Shin et al. (2017) introduced a new filtering algorithm to smooth the single-frequency pseudorange observations of the Android devices. The method is almost not affected by the ionospheric variations. The Hatch filter is the most general filtering algorithm for the GNSS pseudorange smoothing based on the variation of carrier-phase observations. The method can reduce the noise level of the GNSS pseudorange observations but causing an ionosphere-induced error especially for the low-elevation angles satellites. Therefore, Shin et al. (2017) proposed a new single-frequency divergence-free Hatch filter method with the aim of reducing the effect of ionosphere-induced error based on the grid ionospheric vertical error (GIVE) from the SBAS messages. The new method was then applied to the raw measurement of a Nexus 9 device to investigate its efficiency compared with the classical Hatch filter. The root mean square (RMS) of Nexus 9 pseudorange noise was reduced from 5 to 0.6 m for all satellites and the RMS of the horizontal positioning error was less than 1.5 m. Liu et al. (2018) also presented an improved Hatch filter algorithm in the case of duty-cycle existence leading to the positioning accuracy of less than 5 m using the DD pseudorange observations from a Huawei P10 smartphone. Geng et al. (2019) also proposed an improved Hatch filter algorithm called three-thresholds and single-difference Hatch filter (TT-SD Hatch filter) for sub-meter SPP using Android raw GNSS measurements without any need to external augmentation corrections. In this method, the smoothing window width is not fixed and changes considering the thresholds detection for ionospheric cumulative errors, cycle slips and outliers. The results indicated the better performance of the TT-SD Hatch filter method compared with the classical Hatch filter in both static and kinematic tests.

The French Space Agency namely CNES introduced two smartphone applications, the Radio Technical Commission for Maritime (RTCM) converter and PPP WizLite smartphone app (Laurichesse et al., 2017). The first app converts the smartphone measurements to the RTCM format. The smartphone measurements are then transferred to a caster in the well-known RTCM standard for a further use. The positioning software can then be employed to process the stream pulled from the caster. The second application is a port of the CNES PPP-wizard user client allowing undifferenced ambiguity resolution which leads to the centimeter-level positioning accuracy in PPP mode (Laurichesse & Privat, 2015). However, such an accuracy-level cannot be reached using the smartphone measurements. Therefore, Laurichesse et al. (2017) presented a new technique which employs the Doppler filtering and SBAS leading to the sub-meter- and meter-level accuracy in static and kinematic modes, respectively, from the smartphones. Privat et al. (2018) also presented the results of implementing the two Android applications, the raw GNSS measurements convertor to RTCM format and the PPP WizLite from CNES, in both static and kinematic modes. Based on the results, the PPP WizLite app still needs to improve to achieve better positioning accuracy.

Gill et al. (2017) assessed the accuracy of GPS-only single-frequency PPP with a Nexus 9 smartphone by employing the Global Ionospheric Maps (GIM) to account for the ionospheric delay. The results indicated the RMS of 37 cm and 51 cm for the horizontal and vertical components, respectively, using the cellphone.

Riley et al. (2018) have investigated the GNSS measurement and positioning performance of several Android phones/tablets to consider the repeatability of their results. The devices showed significant differences in their tracking performances. At the time of conducting that research, the Broadcom BCM47755 GNSS chipset, which is a dual-frequency GNSS chipset, was developing to use in future smartphones. Introduction of dual-frequency Broadcom chipset (BCM47755) represented a significant milestone in smartphone positioning. Riley et al. (2018) connected this next-generation GNSS chipset to a cell-phone equivalent GNSS antenna and investigated their potential positioning performance obtained from RTK, carrier-phase Trimble RTX and a pseudorange-based solution using the RTX corrections. Trimble CenterPoint RTX is a worldwide service enabling a PPP-like positioning with ambiguity fixing providing centimeter-level accuracy for the real-time applications in static or kinematic modes (Chen et al., 2011). Based on their results, the centimeter-level accuracy could be achieved in both RTK and RTX/PPP solutions in ideal static scenarios.

The above-mentioned researches mostly belong to the single-frequency GNSS smartphones. Since the release of the world’s first dual-frequency GNSS smartphone Xiaomi 8 in May 2018, the researchers have been intensively investigating the performance of the dual-frequency GNSS smartphones. Dual-frequency GNSS smartphones enable the users to make ionospheric-free linear combinations between observations of two frequencies to eliminate the ionosphere effect.

The NSL’s FLAMINGO (Nottingham Scientific Limited’s fulfilling enhanced location accuracy in the mass-market through Initial Galileo Services) team investigated the PPP and RTK performance of the dual-frequency Xiaomi Mi8 smartphone (Fortunato et al., 2019a; Roberts et al., 2018). The results confirmed that the carrier-phase observations from the Xiaomi Mi8 were not affected by the duty cycling and employing the L5/E5a observations could improve the positioning accuracy (Fortunato et al., 2019a; Roberts et al., 2018).

Robustelli et al. (2019) assessed the performance of a Xiaomi Mi8 smartphone in terms of the pseudorange multipath and noise compared with a geodetic receiver using the multipath linear combination. The results indicated a lower C/N_0_ and higher multipath compared with those of the geodetic receiver. Also based on the results, the Galileo measurements had a lower multipath error compared with the GPS ones. The results demonstrated the better quality of the L5/E5 measurements compared with the L1/E1 observations. They also investigated the performance of single point positioning using the Galileo E5a pseudorange observations compared with those of the E1 signal. Robustelli et al. (2021) then assessed the quality of the smartphone observations. The results indicated a low C/N_0_ dependence on satellite elevation and a clear azimuthal asymmetry of signal gain. They also showed the observation noise is different for different devices, constellations, and frequency bands. For instance, the code noise of the second frequency (GPS L5 and Galileo E5a) is less than that of the L1 frequency. They then evaluated the effect of proper stochastic modelling (C/N_0_-dependant weighting model) on the SPP solutions in static mode, caused an improvement in solutions.

Elmezayen and El-Rabbany (2019) investigated the positioning accuracy of the Xiaomi Mi8 smartphone in both post-processing and real-time PPP modes using the combined GPS/Galileo dual-frequency observations. Their numerical results demonstrated that decimeter-level positioning accuracy could be obtained in both post-processing and real-time static PPP modes while meter-level positioning accuracy could be achieved in the kinematic mode.

Wu et al. (2019) also employed the dual-frequency GPS (L1/L5) and Galileo (E1/E5a) observations from a Xiaomi Mi8 smartphone. They have analyzed the positioning performance of the dual-frequency PPP algorithm in both static and kinematic modes. Their numerical results showed that the RMS of the position errors (after convergence to 1 m) was 21.8 cm, 4.1 cm, and 11.0 cm for the East, North, and Up components, respectively, in static mode. However, in kinematic mode, the positioning performance of the PPP algorithm employing the ionosphere-free combination was at the meter-level.

Chen et al. (2019) analyzed the characteristic of raw pseudorange and carrier-phase observations of several GNSS smartphones, Huawei Honor 9, Huawei P10, and Xiaomi Mi8. They also proposed a modified single-frequency PPP algorithm in which separate clock biases for pseudorange and carrier-phase observations are estimated. This is because the fact that the differences between pseudorange and carrier-phase observations of all the three mobile phones are not fixed. Using a Xiaomi Mi8 smartphone, the modified real-time PPP positioning strategy had good performance and the average horizontal and vertical RMS error were 0.81 m and 1.65 m, respectively.

Fortunato et al. (2019b) presented two different real-time applications of smartphones in Geoscience, detecting movements of frequency and amplitude similar to seismic waves and ionosphere monitoring using raw GNSS measurements from a Xiaomi Mi8 smartphone. The results indicated the feasibility of using the Xiaomi Mi8 for real-time ionosphere monitoring as well as fast and periodic movements detection.

Psychas et al. (2019) evaluated the performance of code-only-based SPP and PPP using the raw GNSS dual-frequency measurements of a Xiaomi Mi8 smartphone with a focus on GPS and Galileo only systems within a 14-h time span dataset. They provided static positioning solutions in different cases for example single-frequency uncombined (GPS-only and Galileo-only), combined (GPS + Galileo) models, dual-frequency uncombined and combined models in both real-time and post-processing modes. They then assessed the performance of these solutions in terms of their repeatability and accuracy with respect to the true position of the pillar where the smartphone was placed. It was shown that the dual-frequency GPS + Galileo SPP solution had a better performance compared with the single-frequency uncombined SPP. The PPP solutions were also converged to the sub-meter level accuracy in all different cases. However, based on the results, the combined GPS + Galileo solution resulted in reducing the convergence time to the sub-meter-level horizontally accuracy (less than 4 min).

Guo et al. (2020) analyzed the characteristics of raw GNSS observations from a dual-frequency GNSS smartphone Xiaomi Mi8 in terms of C/N_0_, pseudorange and carrier-phase observations noise, approximate percentage of pseudorange gross errors and carrier-phase cycle slips. They also assessed the performance of a Xiaomi Mi8 smartphone as the navigation tool assuming that only the broadcast ephemeris is available with no link to the reference stations for obtaining observations or to the analysis centers for getting the State Space Representation (SSR) products. To this end, they carried out experiments in both static open-sky and dynamic complex environments. They showed high correlation between the pseudorange noise and the C/N_0_ values and proposed a C/N_0_-dependent weight model for the Xiaomi Mi8. It has also been addressed by several researchers before. Their numerical results also indicated that the noise of the ionosphere-free observations is much larger than the ionospheric delay effects. So, the traditional dual-frequency ionosphere-free combination is not proper for the Xiaomi Mi8 raw GNSS data processing. They then proposed a time differenced (TD) positioning filter to take advantages of the high precision carrier-phase observations. The results indicated that the proposed TD filter algorithm has a satisfying performance especially with the inclusion of the L5/E5 observations.

Aggrey et al. (2020) also investigated the capability and performance of PPP using several smartphones including Xiaomi Mi8, Google Pixel 3, Huawei Mate 20 and Samsung Galaxy S9. Their numerical results indicated the decimeter-level to meter-level horizontal error for both static and kinematic scenarios.

Shinghal and Bisnath (2021) investigated the quality of GNSS measurements of a Xiaomi Mi8 dual-frequency smartphone in different environments. They showed that the carrier-phase measurements suffer from frequent gaps leading to bad positioning results. They then proposed a prediction technique for the data gaps filling as well as a C/N_0_-based stochastic model to introduce a more reliable a priori weights to the observables in the PPP adjustment procedure. The results indicated that employing the proposed measurement prediction model and the new stochastic modeling led to a 64% decrease in the horizontal positioning RMS error for the data collected in suburban areas when the smartphone placed on the car dashboard. A 62% decrease in the positioning error and a 23% increase in positioning availability were also indicated for the high-multipath environments.

Similar to the geodetic receivers, how to model the ionospheric delays of the smartphone GNSS observations plays an important role in high-accuracy positioning. A few recent studies then focused on the effect of ionosphere on the smartphone positioning performance. Banville et al. (2019) considered the impact of different ionosphere modeling by employing ionospheric constraints either precise slant total electron content (STEC) corrections obtained from the GIMs or a regional network of stations. The results indicated that incorporating precise ionospheric information from a regional network could improve the PPP solution, especially when users are located close to reference stations. Wang et al. (2021) proposed the Smart-PPP method employing the uncombined PPP model with the aid of real-time ionospheric vertical TEC (VTEC) products. In this method, two separate clocks are estimated for the code and carrier-phase observations to compensate the inconsistency between the code and carrier-phase observations. Based on the numerical results, the decimeter-level accuracy could be obtained after convergence using the proposed Smart-PPP approach while about sub-meter-level accuracy could be achieved in kinematic mode. Liu et al. (2021) provided a real-time regional ionospheric correction model retrieved from the regional Continuously Operating Reference Stations (CORS) observation data to improve the smartphone positioning accuracy. They then investigated the performance of the proposed method on the real-time carrier-smoothing pseudorange and single-frequency PPP solutions. Employing the proposed method resulted in an improvement in positioning accuracy and a decrease in required convergence time, especially in vertical component, compared to the Klobuchar model. Recently, Yi et al. (2021) investigated the performance of the ionospheric constraints (ionosphere-weighted) PPP model compared to the traditional PPP model using the three different grades of GNSS receivers (geodetic, low-cost, and smartphone hardware) in open-sky and suburban environments. The results indicated that employing the ionospheric constraints is more beneficial to the smartphone PPP solution performance leading to an improvement in horizontal RMS as well as a reduction of the PPP convergence time.

There are also some limited research papers on the PPP Ambiguity Resolution (PPP-AR) using the smartphone observations. For example, Asari et al. (2017) presented the PPP-AR applicability employing the SSR correction data using a smartphone-grade antenna resulting in the sub-meter level positioning accuracy. It should be noted that they used an external survey-grade antenna for their experiment. Wen et al. (2020) also performed the PPP-AR on the Xiaomi Mi8 smartphone observations. However, they used an external survey-grade antenna to replace the Xiaomi Mi8’s embedded GNSS antenna to collect data. Using this enhanced device, the possibility of fixing undifferenced ambiguities with Android dual-frequency GNSS smartphones has been demonstrated. Their numerical results also indicated that centimeter-level accuracy can be obtained performing the PPP-AR method.

#### Relative positioning

In addition to the above-mentioned researches which were mostly related to the single or precise point positioning, there are also several researches applied the relative positioning method to the smartphone GNSS observations. For instance, Realini et al. (2017) presented the accuracy of relative positioning of a smart device with respect to a physical base station (geodetic or low-cost) using the DD carrier-phase observations on the L1 frequency. The decimeter-level accuracy can be obtained through rapid-static surveys with float phase ambiguities using the single-frequency GNSS smartphones, Google & HTC tablet Nexus 9 whose GNSS chip is free from duty cycle, over baselines ranging from approximately 10 m to 8 km. Warnant et al. (2018) evaluated the positioning performances of the Xiaomi Mi8 smartphone. The results indicated that the carrier phase-based static differential positioning using GPS and Galileo provide centimeter- and decimeter-level precision in the horizontal and vertical components, respectively, over a short baseline. Weng et al. (2020) described the derivation of the DGNSS based on the NMEA messages. They then proposed the DGNSS infrastructures that correct the standalone GNSS position of smartphones using the corrections from the reference station. Based on the results, the DGNSS infrastructure can be employed effectively in applications requiring more accuracy without any need to hardware modifications.

Zhang et al. (2019) proposed an optimized multi-GNSS kinematic positioning method called Smart-RTK to improve the kinematic positioning performance with a smartphone. They applied a Doppler-smoothed-code (DSC) filter instead of a carrier-phase smoothed-code filter to reduce the noise level of pseudorange observations. Generally, the carrier-phase observations are used to smooth code measurements. They, however, suffer from frequent cycle-slips when using the smartphones (Zhang et al., 2018). Zhang et al. (2019) employed the Doppler measurements of Android smart devices, which are free of cycle-slips, for code smoothing. They then proposed a constant acceleration (CA) model to predict the kinematic states of smart device users. The results indicated that the smart-RTK method had better performance than the chipset solutions. The RMS error of the horizontal component was 0.3–0.6 m in static mode, 0.4–0.7 m in walking condition and 0.85 m in vehicular experiment.

Paziewski et al. (2019) provided a comprehensive evaluation on the quality of the smartphone observation with a specific focus on the anomalies presented in the carrier-phase and code observations from a GNSS smartphone due to the duty cycling. They showed that the smartphone GNSS observations are affected not only by the high measurement noise and multipath but also by the anomalies such as duty cycling and gradual accumulation of phase errors. They then assessed the medium- to long-range code-based relative positioning and investigated different weighting schemes to find a proper weighting method which considers the low quality of the smartphone GNSS observations. Based on their results, the C/N_0_-dependent weighting scheme was superior to the satellite elevation one.

Dabove and Di Pietra (2019a) evaluated the positioning accuracy of performing NRTK using the single-frequency GPS-only and GPS + GLONASS smartphones measurements considering a CORS network with a mean inter-station distance of 50 km. They showed that the decimeter-level or even centimeter-level accuracy can be obtained through rapid-static surveys without phase ambiguity fixing. Dabove and Di Pietra (2019b) considered the positioning performances of the dual-frequency Xiaomi Mi8 smartphones over a single-baseline RTK positioning with a geodetic receiver or a smartphone as the master (reference) device. Based on their numerical results, a cm-level of precision and sub-meter level 3D accuracy was achieved even when a smartphone was considered as the master station. However, it was not possible to fix the carrier-phase ambiguities to their integer values.

Various researches have been also conducting to investigate the feasibility of ambiguity resolution with a smartphone receiver, either using an external GNSS antenna or using the smartphone antenna itself. Håkansson (2018) investigated the characteristics of the DD carrier-phase ambiguities of a Nexus 9 tablet and concluded that the carrier-phase ambiguities cannot be estimated as integers from the Nexus 9 measurements. Li and Geng (2019) then explained the cause of this phenomenon, which is caused by unaligned initial phase bias (IPB). They also analyzed the characteristics of the raw GNSS measurements from the smartphones and refined the error model. Based on their results, the GNSS signals from the smartphones do not have uniform and consistent signal strength. They also observed rapid changes in C/N_0_ values and low C/N_0_ even at the high elevation angles. They then considered the positioning performance of the GNSS smartphones using the relative positioning method as well as the SPP method. Using the carrier-phase relative positioning solutions of Nexus 9, the centimeter-level and decimeter-level precision could be obtained in the static and kinematic modes, respectively. However, the positioning accuracy of the RTK solutions using the GPS and GLONASS observations is worst compared to the GPS-only since the GLONASS pseudorange noise is 3–4 times larger than that of the GPS one. Geng and Li (2019) later investigated the feasibility of resolving Android GNSS carrier-phase ambiguities using smartphones connected to external survey-grade antennas. They found an unaligned chipset IPBs within the Android carrier-phase data. Calibrating IPB allows the recovery of the integer nature of carrier-phase ambiguities resulting in about 30%–80% improvement in positioning accuracy compared with the ambiguity-float solutions. Paziewski et al. (2021) also investigated the feasibility of integer ambiguity resolution by computing the DD phase residuals of smartphones. Based on the results, the DD phase residuals suffers from unwanted effects (long-term drift) and noise caused not preserving the integer nature of ambiguities. However, such a phenomenon was not observed in the phase observations of Xiaomi Mi8. Gao et al. (2021) first introduced a new stochastic model for the pseudorange observations, called the raw observation standard deviations (ROSTDs), based on the “Received Time UncertaintyNanos” variable from the Android API. They then investigated the ambiguity integer property by analyzing the residuals of DD carrier-phase observations between a smartphone and a high-end geodetic receiver. They realized that the integer property of carrier-phase observations of the tested devices cannot be possessed, except for the Huawei P30 and Xiaomi Mi8 devices after a linear fitting to restore the integer property of phase ambiguities (detrending).

There are also some attempts to determine the smartphone antenna characteristics. For example, Netthonglang et al. (2019) attempted to determine the Xiaomi Mi8’s GNSS antenna phase center by averaging the post-processing coordinates in northing and easting. 

They found that the Xiaomi Mi8 phase center is located on the top left of the device (about 2.8 cm and 0.9 cm from left and top, respectively). Bochkati et al. (2020) attempted to determine the antenna phase center of the three different Xiaomi Mi8 devices, showing different location for them. It indicates that the antenna phase center may not be the same even for the devices of the same model. Wanninger and Heßelbarth (2020) later performed a relative calibration to retrieve the antenna phase center offset and variation (Phase Center Offset (PCO) and Phase Center Variations (PCV)) of a Huawei P30 device for the L1 frequency. They analyzed the GNSS observations of the dual-frequency GNSS chip Kirin 980 embedded into Huawei P30 using more than 80 h of static observations at different locations. They processed the code and carrier-phase observation in relative positioning mode with respect to a geodetic-grade device. Employing only GPS L1 observations, they could fix the carrier-phase ambiguities considering the estimated PCO and PCV for the L1 frequency. Their results indicated that the 3D position errors (standard deviations) of a few centimeters and 2 cm can be obtained after 5 min and for longer observation session, respectively. They also stated that reliable ambiguity-fixing cannot be done on the other signals since they do not possess integer properties. An accurate antenna calibration requires a large number of observations and the resolved carrier-phase ambiguities to their true integer values (Heßelbarth & Wanninger, 2020). Therefore, Heßelbarth and Wanninger (2020) investigated whether the carrier-phase observations possess the property of integer ambiguities by computing the DD residuals over a short and known baseline to a GNSS reference station. They showed that not all carrier-phase observations have the property of integer ambiguities. Darugna et al. (2019) showed that it is not possible to successfully perform ambiguity resolution because of the residual phase biases caused by the multipath. Subsequently, Darugna et al. (2021) has performed an absolute, multi-frequency (L1 and L5) antenna calibration for the dual-frequency Huawei Mate20X smartphone employing the robot-based absolute antenna field calibration. They then reported an improvement in smartphone positioning performance after applying the antenna corrections, showing a cm-level 2D RMS with successful ambiguity resolution, especially in positioning in open sky environment. Thanks to the new update of Google starting in Android 11 (API 30), one can access to the antenna characteristics of the smart device (i.e., PCO and PCV corrections) through the GnssAntennaInfo class. However, those corrections are only corresponded to the device model, and not an individual device.

**Fig. 12:**
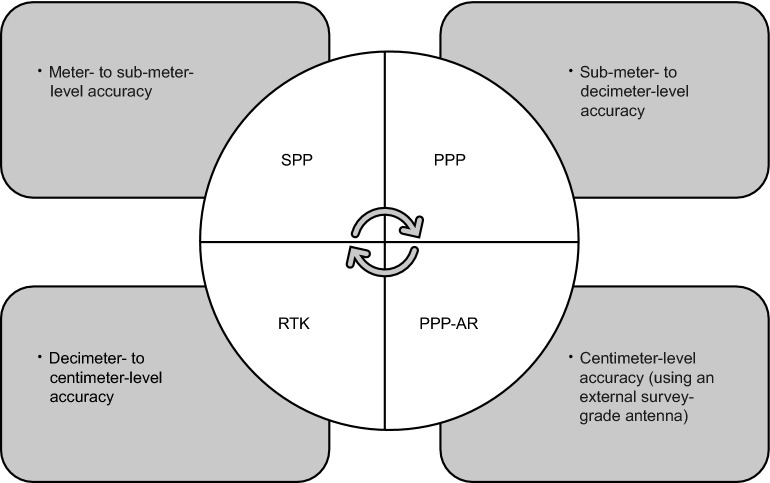
Summary of achievable smartphone positioning accuracy reported in research papers

To improve the positioning performance, the fusion of other navigation sensors such as inertial measurement units (IMU) with the GNSS chipset can be also considered which is the subject of the next subsection. Before starting the next section, a summary of achievable smartphone positioning accuracy using different methods is provided in Fig. 12. We should note that what were reported as the achievable accuracy depend on different factors such as the environment and the positioning mode (static and kinematic) and it is not unique. Table 5 also gives the pros and cons of each method. Recently, Shinghal and Bisnath (2021) compared the positioning accuracy and availability of dual-frequency PPP, RTK and the internal positioning solution of a Xiaomi Mi 8 using a kinematic dashboard dataset in suburban environments. The results revealed the RTK method had better performance in terms of accuracy while their post-processed PPP solution outperformed RTK in terms of the solution availability. In addition, both PPP and RTK solutions were more accurate than the internal positioning solution.

**Table 5:** Pros and cons of smartphone positioning using different methods

Method	Pros	Cons
SPP	Simple and straightforward method Only observations of a single device needed No need to additional data/corrections	Noisy pseudorange measurements Affected by multipath Low accuracy
PPP	Only observations of a single device needed Higher accuracy compared to SPP	Frequent cycle slips and missing phase observations Precise satellite orbit and clock needed Unknown antenna characteristics of the smart devices (PCO and PCV) Long convergence time
PPP-AR	Higher accuracy	An external survey-grade antenna needed (Wen et al., 2020)
RTK	Higher accuracy Allows integer ambiguity resolution	Base station(s) needed Not all carrier-phase observations have the property of integer ambiguities

#### GNSS/INS integration

Inertial sensors are also embedded inside the latest smartphones which are mostly MEMS (micro-electro-mechanical sensors) based IMU consisting of three mutually orthogonal accelerometers and three orthogonal Gyroscopes which measure linear acceleration and angular velocity, respectively. The IMU sensors can be integrated with the GNSS observables to achieve a better localization solution. Sheta et al. (2018) employed the raw GNSS measurements and the inertial sensors data from the smartphones to improve positioning solution. They used the Huawei mate 8 as a testing platform and investigated the accuracy of inertial sensors only solution and loosely coupled GPS/INS integration solution. The results indicated that the GPS/INS integration solution is better compared to the INS-only solution. However, they only provided the positioning error for the East component using only 45 s of data. Also, the constellations they used was not mentioned and they only stated that the GPS data provided in the NMEA format was employed. Mostafa et al. (2019) used the integration of GNSS, an INS-smartphone and other visual sensors to enhance the USV (unmanned surface vehicle) navigation system, with around 80% reduction in errors. Yan et al. (2019) provided an initial performance assessment of the Android smartphone’s IMU in a GNSS/INS coupled navigation model. They also investigated the quality of raw IMU data from two smartphones “Xiaomi Mi8” and “Honor Play” by comparing their records with a higher grade IMU's records (reference IMUs) through two kinematic tests. A good matching was observed between the IMU data derived from the smartphones and the reference IMUs. Niu et al. (2019) combined RTK with an IMU-based pedestrian navigation algorithm to assist RTK and to improve positioning performance in urban areas. Their experiments confirmed the feasibility of the proposed method to provide continuous and robust positioning results in GNSS-challenged environments with a Xiaomi Mi8 smartphone. Yan et al. (2020) first showed that the gyro and accelerometers records from the smartphones have different sampling intervals. They then proposed a modified Kalman filter to consider all IMU data with different sampling rates through the coupled GNSS/IMU integration algorithm. The results indicated a significant improvement in a simulated GNSS denied. Bochkati et al. (2020) aimed to the stochastic modelling of the smartphone inertial sensors measurements using the Allan variance method. They showed that the built-in MEMS IMU inside the Xiaomi Mi8 smartphone has relatively reliable and steady behaviour compered to a commercial MEMS device. In addition, the results indicated that the contribution of the IMU measurements could not improve the success rate of the RTK carrier-phase ambiguity fixing and the Xiaomi Mi8 smartphone could only provide float solution with meter-level accuracy, even in the case of loosely-coupled GNSS/INS integration.

## Smartphone positioning challenges and future perspectives

Despite a lot of efforts devoted to the smartphone positioning, the GNSS smartphone positioning is still in its early stage. There are still several major challenges in the following aspects: (1) smartphone GNSS observations, (2) smartphone/device, (3) environment effect and smartphone holding modes and (4) algorithm development. They are briefly explained below:

- Smartphone GNSS observations: The smartphone GNSS observations are very noisy since they use the cellphone-grade GNSS chipsets and antennas. Such ultra-low-cost GNSS chipsets and antennas have lower gain resulting in low and irregular C/N_0_. This also makes it more difficult to distinguish the direct line of sight (LOS) signals from the non-line of sight (NLOS) signals where the latter would result in large multipath effect on the GNSS observations. Another issue regarding the smartphone GNSS observations is that the carrier-phase observations suffer from the frequent cycle slips and missing of phase observations.
- Smartphone/device: For PPP, the antenna characteristics of the smart devices (i.e., PCO and PCV corrections) must be precisely known. The PCO and PCV corrections are now available through the GnssAntennaInfo class added in API 30 (Android 11). However, those corrections are only corresponded to the device model, and not an individual device.
- Environment effect and smartphone holding modes: Most of the researches have been carried out in open-sky and low-multipath environments, so there is little understanding on the performance of smartphone positioning in harsh environment such as urban areas that are more vulnerable to the multipath and/or satellite obstructions. It is therefore essential to investigate the performance of smartphone positioning in such environments where most phones are used. Furthermore, how to hold and use the smartphone could significantly affect the positioning performance. For example, is the phone held in your hand or in your pocket while walking?
- Positioning algorithm development: Despite great improvement in algorithm development of smartphone positioning, new algorithms are needed to address those mentioned challenges. For instance, most researches have provided only post-processed results. Therefore, real-time high-accuracy positioning using smartphones should be further investigated.

The rapid increase in number of GNSS smartphone consumers around the world provides a great opportunity not only for the academic sector but also for the industrial sector. Besides the researches focusing on the high-accuracy positioning using the mass-market devices, industrial companies are also interested in this field. Industry experts expect that the mass-market devices would become widely applicable to the high-accuracy applications in the future. They believe that fully autonomous navigation needs a horizontal positioning accuracy of 20–30 cm. It will therefore be expected that fully autonomous navigation becomes a possible application of positioning with mass-market devices. Industry experts also believe that in addition to the accuracy, the convergence time should be considered so that the users expect to use a (near) real-time precise positioning system (European GNSS Agency, GSA, 2019b). As an example, the Geo++ Company is one of the industrial companies interested in smartphones positioning as they have planned to develop RTK on the smartphones (Francesco et al., 2019). However, it is still in its early stages and limited information on the status of the project is available.

Even if the accuracy of the smartphone positioning is not at the same level with those of the high-end geodetic receivers, the meter- and sub-meter-level accuracy obtained from the smartphones meet the required accuracy for a wide range of applications such as mapping and GIS (Geographic Information Systems), pedestrian and vehicle navigation, autonomous navigation, object tracking, traffic monitoring and planning, social networking, mobile-location based apps, safety and emergency, games and sports (van Diggelen et al., 2018). European GSA (2018b) also classified applications which can benefit from higher positioning accuracy of the smartphones into three categories as follows:

1. Mobile applications such as location-based advertising and augmented reality (AR)
2. Safety related applications such as GNSS-enabled emergency call and mobile health
3. Semi-professional applications such as mapping, workforce management and smart city asset management

Although the current accuracy of the smartphone positioning may be sufficient for the mentioned applications, we will still take advantage of improving smartphone positioning accuracy. The higher positioning accuracy from the mass-market mobile devices, the more uses of them in (semi-) professional applications. With improvements in new generation of GNSS chipsets and the smartphone positioning algorithms, the smartphone positioning accuracy is expected to improve in the near future.

## Concluding remarks

Thanks to the new API implemented on Android 7 or later, the use of smartphones for most applications such as cadastral surveying, mapping surveying applications and navigation has been increasing due to the cost-effectiveness of the GNSS smartphones. However, there are still some limitations in the high-precision smartphone positioning. They include the low quality of the GNSS smartphones measurements, their highly susceptibility to the multipath error due to the linear polarization structure of the smartphone antennas, frequent cycle slip and missing phase observations, and lack of phase center offset and variation information for most smartphone GNSS antennas. These limitations restrict the users to achieve high-precision smartphone positioning. Therefore, there is a high demand on the development of new methods and algorithms to improve smartphone positioning accuracy and reliability as well as the development of new smartphone-based precision applications.

## Data Availability

The datasets analyzed during the current study are available at https://www.kaggle.com/c/google-smartphone-decimeter-challenge/data.

## Footnotes

1 https://barbeau.medium.com/.
